# Relationship between thyroid hormone levels and metabolic dysfunction associated steatotic liver disease in patients with type 2 diabetes: A clinical study

**DOI:** 10.1097/MD.0000000000038643

**Published:** 2024-06-28

**Authors:** Tingbo Bi

**Affiliations:** a Department of Clinical Laboratory, the First Affiliated Hospital of Jinzhou Medical University, Jinzhou City, Liaoning Province, China.

**Keywords:** autophagy in liver, clinical diagnosis, diabetes mellitus thyroid function liver diseases, endocrinological disorders, lipid metabolism, steatosis insulin resistance, treatment strategies

## Abstract

**Background:**

This study investigates the correlation between thyroid hormone levels and metabolic dysfunction in patients with type 2 diabetes mellitus (T2DM) who exhibit normal thyroid function and metabolic dysfunction associated with steatotic liver disease (MASLD).

**Objective:**

The objective is to identify a scientific basis for the management of T2DM complicated by MASLD, aiming to refine clinical strategies and enhance patient well-being.

**Methods:**

Statistical analysis was conducted using SPSS 26.0, employing independent sample t-tests for normally distributed data and logarithmic transformations for non-normal data to meet analysis prerequisites. Multifactorial logistic regression analysis elucidated the impact of various factors on the risk of MASLD in T2DM patients.

**Results:**

Elevated levels of FT3 may be associated with an increased risk of nonalcoholic fatty liver disease. Additionally, the FT3/FT4 ratio has been validated as an effective serological marker for predicting the risk of MASLD. In patients with DM2 and normal thyroid function, changes in thyroid hormone levels are closely related to the occurrence of MASLD. Elevated levels of FT3, total triiodothyronine (TT3), and thyroid-stimulating hormone are associated with an increased risk of MASLD.

**Conclusion:**

FT3, TT3, and thyroid-stimulating hormone have important clinical value in the diagnosis of patients with T2DM complicated with MASLD.

## 1. Introduction

Recent studies have significantly advanced the understanding of nonalcoholic fatty liver disease (NAFLD) among scholars across various fields, particularly in terms of disease nomenclature. In June 2023, several authoritative societies released the Delphi consensus statement, officially introducing the term “Metabolic dysfunction associated steatotic liver disease (MASLD).”^[1]^ This term is designated to describe adults who are overweight or obese, as well as individuals with type 2 diabetes mellitus (T2DM) or at least 2 abnormal metabolic risk factors, confirmed to have liver steatosis through imaging, serological, or histological examinations. With the naming of MASLD, the associated diagnostic criteria have also been updated to reflect a deeper understanding of the disease.

Historical literature often associates hypothyroidism closely with NAFLD.^[2]^ However, some retrospective cohort studies indicate no direct link between hypothyroidism and the incidence of NAFLD.^[3]^ A 2020 study exploring the obese population found an increased risk of NAFLD among individuals with higher levels of thyroid-stimulating hormone (TSH) and triiodothyronine (T3), based on serological markers.^[4]^ Furthermore, individuals with both T2DM and NAFLD exhibited higher TSH levels, lower thyroxine (T4) levels, and a higher positivity rate for antithyroid peroxidase antibodies compared to non-NAFLD patients.^[5]^ Notably, current medical research reports are scarce regarding the relationship between thyroid hormone levels and the risk of MASLD in patients with T2DM who have normal thyroid function. The paucity of data in this research area suggests that, despite the recognized link between thyroid hormones and metabolic diseases, the specific mechanisms by which thyroid hormone levels affect the risk of MASLD in patients remain unclear. Therefore, this study is dedicated to exploring the potential link between thyroid hormone levels and the risk of MASLD among patients with DM2 in the west region of China with normal thyroid function. The primary objective is to determine whether thyroid hormone levels can serve as biomarkers for predicting the risk of MASLD, thereby providing more information for the clinical management of patients with T2DM.

## 2. Methods

### 2.1. Study population

This study conducted data collection from January to December 2023 on 284 patients diagnosed with T2DM at the endocrinology and metabolism of the First Affiliated Hospital of Jinzhou Medical University. All participants were over the age of 18. Within this cohort, 194 patients were diagnosed with steatotic liver disease, while the remaining 90 were not diagnosed with MASLD. The criteria for normal thyroid function within this study were based on the following reference ranges for thyroid hormones: TSH levels ranging from 0.34 to 5.60 μU/L and free thyroxine (FT4) levels ranging from 0.58 to 1.64 ng/dL. These standards were used to ensure consistency in thyroid function among subjects, facilitating a more accurate assessment of the correlation between thyroid hormone levels and the risk of MASLD.

### 2.2. Measure

During the medical history collection process, healthcare professionals adhere to standardized procedures to gather personal information from patients. This includes, but is not limited to, basic demographics such as age, gender, and educational level. Additionally, a detailed record of the patient’s medical history is maintained, encompassing any diabetes-related complications and lifestyle factors, such as smoking and alcohol consumption habits. On the first day of admission, clinical data collection is performed by measuring the patient’s height, weight, and blood pressure to calculate the body mass index (BMI).

### 2.3. Biochemical determinations

On the morning following admission, patients underwent fasting venous blood collection. The serum was then separated by centrifugation and a comprehensive biochemical analysis was performed using the ACCELERATOR α3600 automated biochemistry analyzer. The parameters measured included high-density lipoprotein cholesterol (HDL-C), low-density lipoprotein cholesterol (LDL-C), triglycerides (TAG), total cholesterol (TC), glutamic pyruvic transaminase (GPT), glutamic oxaloacetic transaminase (GOT), gamma-glutamyl transferase (γ-GT), and thyroid function indicators: thyroid stimulation hormone (TSH), thyroglobulin antibody (TGAb), free triiodothyronine (FT3), FT4, and thyroid peroxidase antibody (TPOAb). Furthermore, the levels of glycated hemoglobin (HbA1c) were determined using the Premier Hb9210 analyzer to assess glycemic control. These measurements provided vital biochemical and endocrinological parameters for subsequent data analysis.

### 2.4. Statistical analysis

The study used SPSS 26.0 software for data analysis. Initially, all observed indicators were subjected to normality tests. Continuous variables that conformed to a normal distribution were expressed as (x ± s) and compared between groups using independent sample *t* tests. For continuous variables that do not follow a normal distribution, the median and interquartile range (M(Q1*–*Q3)) were used, with the Mann–Whitney *U* test comparing the medians between groups. Qualitative data were presented as percentages and compared between groups using the chi-square test.

Subsequently, univariate logistic regression analysis was used to explore the correlation between each variable and MASLD. A multivariate logistic regression model was also applied to analyze the impact of multiple independent variables on a binary classification factor, delving into the relationship between FT3, FT4, and their relationship with MASLD. Moreover, the odds ratios (OR) and their 95% confidence intervals (CI) for each independent variable were calculated. The odds ratio is a measure of the strength of association between variables, while the confidence interval provides a range of reliability for this estimate. An association is generally considered significant when the confidence interval does not include it. For the determination of significance, a *P* value threshold of .05 was established, which means that if a *P* value less than .05 was obtained, the observed association or effect was considered statistically significant, indicating that the result is unlikely due to chance. Finally, to assess the efficacy of the FT3/FT4 ratio in predicting the risk of NAFLD, receiver operating characteristic (AUROC) analysis was conducted.

### 2.5. T2DM diagnosis

Inclusion and exclusion criteria were implemented according to the guidelines outlined in the “China Type 2 Diabetes Prevention and Treatment Guidelines (2020 Edition).”^[6]^

### 2.6. MASLD diagnosis

According to the multi-society Delphi consensus statement on the new nomenclature for fatty liver diseases,^[7]^ a diagnosis of steatotic liver disease can be established through imaging or histology, and must meet any one of the following 5 criteria:

1. BMI of ≥25 kg/m^2^ (≥23 kg/m^2^ for Asians) or waist circumference of >94 cm for men and >80 cm for women, with variations between different ethnicities.
2. Fasting blood glucose levels of ≥100 mg/dL (≥5.6 mmol/L), 2-hour post-load glucose levels of ≥140 mg/dL (≥7.8 mmol/L), HbA1c levels of ≥5.7%, or undergoing treatment with specific drugs.
3. Blood pressure readings of ≥130/85 mm Hg or treatment with specific antihypertensive medications.
4. Plasma levels of TAG 150 mg/dL (1.70 mmol/ L) or treatment with specific lipid modifying agents.
5. Plasma high-density lipoprotein (HDL) cholesterol levels of <40 mg/dL (<1.0 mmol/L) for men and <50 mg/dL (<1.3 mmol/L) for women or undergoing treatment with specific medications.

## 3. Result

### 3.1. Baseline characteristics of the study population

A comparative analysis was performed between 194 patients diagnosed with T2DM and concurrent MASLD, and 90 patients without MASLD. The results indicated significant statistical differences in the MASLD group for the following parameters: sex (*P* = .048), age (*P* < .001), BMI (*P* < .001), waist-to-hip ratio (*P* < .001), fatty liver (*P* < .001), FT3 (*P* = .001), ratio (*P* = .02), triacylglycerol (*P* < .001), GPT (*P* = .001) and GOT (*P* = .039). However, no significant statistical differences were observed between the 2 groups for TSH, FT4, TGAb, TPOAb, TC, HDL-C, LDL-C, γ-GT, HbA1c, and insulin autoantibodies (IAA), as shown in Table 1.

**Table 1:** Descriptives.

Variable	Total (n = 284)	MASLD (n = 194)	Non-MASLD (n = 90)	*P*
Sex				
Man	163 (57.4)	119 (61.3)	44 (48.9)	.048
Woman	121 (42.6)	75 (38.7)	46 (51.1)	
Age (yr)	56.5 ± 13.9	54.5 ± 14.2	60.9 ± 12.2	<.001
BMI (kg/m^2^)	25.6 ± 3.9	26.6 ± 3.7	23.4 ± 3.6	<.001
Waist-to-hip ratio	0.9 ± 0.1	1.0 ± 0.1	0.9 ± 0.1	<.001
Fatty liver				
Yes	194 (68.3)	194 (100)	0 (0)	<.001
No	90 (31.7)	0 (0)	90 (100)	
TSH (μU/mL)	2.0 ± 1.1	2.0 ± 1.1	2.0 ± 1.1	.779
FT3 (pmol/L)	4.1 ± 0.7	4.2 ± 0.7	3.9 ± 0.7	.001
FT4 (pmol/L)	13.9 ± 1.8	14.0 ± 1.8	13.8 ± 1.7	.43
FT3/FT4 ratio	0.3 ± 0.1	0.3 ± 0.0	0.3 ± 0.1	.02
TGAB (U/mL)	14.0 ± 70.8	12.0 ± 73.1	18.5 ± 65.6	.544
TPOAB (U/mL)	39.5 ± 160.5	27.8 ± 134.2	65.6 ± 206.5	.118
TC (mmol/L)	5.2 ± 1.3	5.2 ± 1.4	5.1 ± 1.3	.475
TAG (mmol/L)	2.4 ± 2.2	2.8 ± 2.5	1.6 ± 0.9	<.001
HDL (mmol/L)	1.1 ± 0.3	1.1 ± 0.3	1.2 ± 0.3	.071
LDL (mmol/L)	3.0 ± 1.1	3.0 ± 1.1	3.2 ± 1.1	.246
GPT (U/L)	28.7 ± 31.1	32.7 ± 34.7	20.2 ± 18.8	.001
GOT (U/L)	22.2 ± 18.2	23.7 ± 20.1	19.0 ± 12.6	.039
γ-GT (U/L)	43.2 ± 50.8	45.0 ± 45.7	39.3 ± 60.5	.385
HBA1C (%)	9.5 ± 2.3	9.6 ± 2.3	9.3 ± 2.1	.391
IAA	10.4 ± 16.9	10.9 ± 18.4	9.6 ± 13.8	.626

BMI = body mass index, FT3 = free triiodothyronine, FT4 = free thyroxine, GOT = glutamic oxaloacetic transaminase, GPT = glutamic pyruvic transaminase, HBA1C = glycated hemoglobin, HDL = high-density lipoprotein cholesterol, IAA = insulin autoantibodies, LDL = low-density lipoprotein cholesterol, TAG = triglycerides, TC = total cholesterol, TGAb = thyroglobulin antibody, TPOAb = thyroid peroxidase antibody, TSH = thyroid stimulating hormone, γ-GT = gamma-glutamyl transferase.

### 3.2. Multivariate logistic regression analysis of serum FT3 Levels and FT3/FT4 ratio in relation to the risk of MASLD

Three multifactorial logistic regression models were designed to evaluate the association of serum FT3 levels and the FT3/FT4 ratio as independent variables, with the presence of metabolic-associated liver disease as the dependent variable. Model 1 analysis revealed a significant positive correlation between elevated FT3 levels and increased risk of MASLD (*P* = .001), with the FT3/FT4 ratio also showing a positive correlation with MASLD risk (*P* = .022). In Model 2, after adjusting for age and gender, the results still indicated a positive correlation between FT3 levels (*P* = .008) and the FT3/FT4 ratio (*P* = .039) with the risk of MASLD. Model 3, based on Model 2, further adjusted for BMI, triacylglycerol, HDL-C, LDL-C, GPT, GOT, and γ-GT, demonstrating that only FT3 levels were independently associated with the risk of MASLD (*P* = .016), as detailed in Table 2.

**Table 2:** Independent risk factors.

Variable	OR	95% CI	*P*
FT3			
Model1	0.52	0.35 to 0.77	.001
Model2	0.57	0.37 to 0.86	.008
Model3	0.54	0.32 to 0.89	.016
FT3/FT4 ratio			
Model1	0	0 to 0.43	.022
Model2	0.01	0 to 0.77	.039
Model3	0.03	0 to 8.2	.212

FT3 = free triiodothyronine, FT4 = free thyroxine.

### 3.3. Prevalence of MASLD across low, medium, and high levels of FT3, FT4, and FT3/FT4

**Figure 1.**
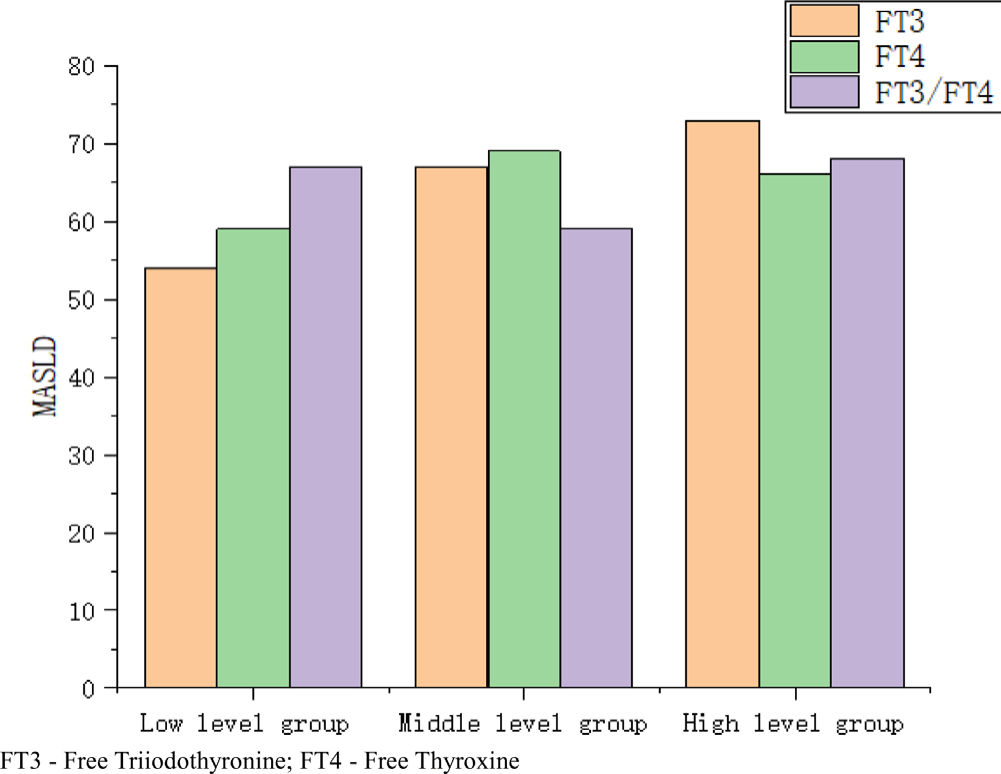
Relationship between different levels of FT3FT4 and FT3/FT4 and MASLD. FT3 = free triiodothyronine, FT4 = free thyroxine, MASLD = metabolic dysfunction associated steatotic liver disease.

In this study, we classified serum FT3 and FT4 levels, as well as the FT3/FT4 ratio, into tertiles to investigate the relationship between these biochemical markers and the prevalence of MASLD. The tertiles for FT3 were defined as <3.92 pmol/L, 3.92–4.41 pmol/L, and >4.41 pmol/L; for FT4, the tertiles were <13.22 pmol/L, 13.22–14.4 pmol/L, and >14.4 pmol/L; and for the FT3/FT4 ratio, the tertiles were <0.28, 0.28–0.32, and >0.32. The results demonstrated a significant increase in the prevalence of MASLD with higher levels of FT3 (Fig. 1), suggesting that elevated levels of FT3 may serve as an early warning indicator for the increased risk of MASLD. Specifically, a higher tertile of FT3 levels was associated with a higher likelihood of MASLD.

### 3.4. Predictive value of the FT3/FT4 ratio for the risk of MASLD

**Figure 2.**
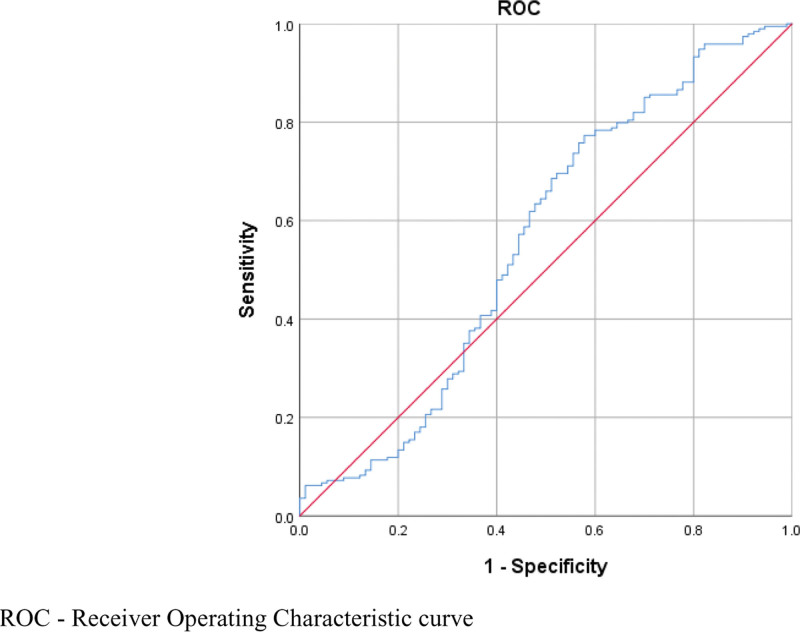
ROC curve analysis of the predictive efficacy of the FT3/FT4 ratio for the risk of MASLD. FT3 = free triiodothyronine, FT4 = free thyroxine, MASLD = metabolic dysfunction associated steatotic liver disease , ROC = receiver operating characteristic.

The study used receiver operating characteristic (ROC) curves to assess the efficacy of the FT3/FT4 ratio to predict the risk of MASLD among patients with T2DM. The results indicate that the FT3/FT4 ratio can effectively predict the likelihood of MASLD in T2DM patients. The area under the curve (AUC) value was 0.586, suggesting that the model has a certain predictive capacity, although not highly efficient. The 95% confidence interval (CI) ranged from 0.527 to 0.645, providing an assessment of the predictive stability. This wide confidence interval implies that despite the model’s demonstrated predictive power, there is still some uncertainty (*P* = .003). The optimal cutoff value was determined to be a FT3/FT4 ratio of 0.272, with a sensitivity of 76.8% and a specificity of 55.3%. This indicates that the model can identify actual patients with MASLD with a 76.8% accuracy rate and exclude non-MASLD patients with a 55.3% accuracy rate, suggesting that the model performs well in identifying MASLD patients but is average in excluding non-patients (Fig. 2).

## 4. Discussion

In previous research on thyroid hormones and liver disease, the focus has predominantly been on studies of NAFLD. However, with the introduction of the term “metabolic dysfunction-associated fatty liver disease” (MAFLD), the applicability of past conclusions to MAFLD remains unverified. Therefore, in the discussion section, this paper will refer to past research conclusions as NAFLD while addressing the findings of this study as MAFLD.

MASLD has emerged as a significant public health issue that affects multiple systems. With the increasing prevalence of obesity, the clinical and economic burden of MASLD is expected to rise.^[8,9]^ It is estimated that MAFLD may become the leading cause of end-stage liver disease in the future. Despite this, the precise mechanisms of MASLD remain unclear. It is associated with central obesity, adiposity, diabetes, hypertension, and other diseases, manifesting as a component of metabolic syndrome.^[9]^

In the previous nomenclature of metabolic associated fatty liver disease, formerly known as NFLD, it is characterized as a hepatic manifestation of metabolic syndrome with specific liver–pancreatic insulin resistance.^[10]^ Thyroid hormones play a pivotal role in the regulation of insulin signaling and glucose homeostasis, which are crucial for metabolic equilibrium.^[11]^ Previous research has established the critical role of thyroid hormones in modulating lipid and carbohydrate metabolism, energy homeostasis, and neurodevelopment. Notably, thyroid hormones significantly influence the synthesis and metabolism of hepatic fatty acids and cholesterol, particularly in maintaining liver homeostasis. Studies indicate that patients with subclinical hypothyroidism exhibit a significantly higher prevalence of NAFLD compared to those with normal thyroid function, with TSH levels independently associated with NAFLD.^[12]^ Research by Lin et al suggests that patients with MAFLD tend to be older than those with NAFLD.^[13]^ The study reveals an increase in the prevalence of NAFLD among women in the past decade, with a notable increase in mortality rates compared to men.^[14]^ Ma and colleagues have demonstrated that the use of levothyroxine can significantly reduce hepatic lipid content in patients with type 2 diabetes and NAFLD with normal thyroid function.^[15]^ Sinha research indicates that thyroid hormone T3 treatment can enhance autophagic and lysosomal activity in hepatocytes, increasing the transport of fatty acids to mitochondria for β-oxidation, a process dependent on thyroid hormone receptors.^[16]^ Therefore, this lipophagic action may alleviate liver lipid deposition, offering protection in NAFLD.^[17]^ Xin et al^[18]^ study finds that in NAFLD patients, a reduction in thyroid hormone-mediated autophagy contributes to the development of hepatic lipid metabolism and steatosis.

The findings of this study reveal a significant association between FT3 levels and the FT3/FT4 ratio with metabolic dysfunction associated fatty liver disease. Specifically, FT3 levels are independently and positively correlated with the risk of MASLD, with an increasing prevalence of MASLD observed alongside rising FT3 levels. The analysis of the receiver operating characteristic curve indicates that the FT3/FT4 ratio serves as an effective serological marker to predict the risk of MASLD, particularly in patients with DM2. However, more research is required to enhance the predictive power and specificity of the model. A review of previous studies notes that in a cohort of 878 elderly Chinese individuals, the prevalence of NFLD decreased with higher serum FT4 levels.^[19]^ Furthermore, a cohort study found that higher serum FT3 levels were independently associated with the prevalence of NAFLD diagnosed based on the fatty liver index, after adjusting for age and gender.^[20]^ However, a systematic review and meta-analysis reported no significant differences in FT3, FT4, and TSH levels between NAFLD patients and controls.^[21]^ The inconsistency in these findings may be attributed to variations in study design, population characteristics, definitions of thyroid dysfunction, and differences in NAFLD diagnostic criteria in the literature.

## 5. Conclusion

In summary, we discovered an independent positive correlation between FT3 levels and the FT3/FT4 ratio with the risk of NFLD in patients with T2DM who have normal thyroid function. Furthermore, the FT3/FT4 ratio has been validated as an effective serological marker to predict the risk of metabolic dysfunction associated fatty liver disease. However, due to the cross-sectional study design employed, we could not further verify a causal relationship between elevated FT3 levels and FT3/FT4 ratio and increased risk of MASLD. Future research, through longitudinal cohort studies or by increasing sample sizes, is needed to strengthen the evidence base for these findings.

Furthermore, this study did not account for factors that might affect thyroid-related indices, such as medication use, diabetes, chronic complications, cardiovascular disease, and other conditions that can interfere with thyroid function, which is one of the limitations of this study. These factors require further investigation and analysis in future research.

Lastly, the absence of diverse ethnicities in the study may affect the relationship between thyroid hormone levels and MASLD. The criteria for normal thyroid function may vary by study, influencing the selection of subjects and affecting the final results. The methods for diagnosing MASLD are varied, including serological tests and imaging assessments, and different diagnostic tools and criteria may lead to variations in research conclusions. Therefore, when applying research findings to clinical practice, physicians need to consider factors such as ethnic differences, definitions of thyroid function, and choices of diagnostic methods. These factors could affect the interpretation and application of research conclusions.

## Author contributions

**Conceptualization:** Tingbo Bi.

**Data curation:** Tingbo Bi.

**Formal analysis:** Tingbo Bi.

**Funding acquisition:** Tingbo Bi.

**Investigation:** Tingbo Bi.

**Methodology:** Tingbo Bi.

**Project administration:** Tingbo Bi.

**Resources:** Tingbo Bi.

**Software:** Tingbo Bi.

**Supervision:** Tingbo Bi.

**Validation:** Tingbo Bi.

**Visualization:** Tingbo Bi.

**Writing – original draft:** Tingbo Bi.

**Writing – review & editing:** Tingbo Bi.
